# Hyena architecture enables fast and efficient protein language modeling

**DOI:** 10.1002/imo2.45

**Published:** 2024-12-07

**Authors:** Yiming Zhang, Bian Bian, Manabu Okumura

**Affiliations:** ^1^ Department of Information and Communications Engineering School of Engineering, Institute of Science Tokyo Yokohama Japan; ^2^ Department of Computational Biology and Medical Sciences, Graduate School of Frontier Sciences The University of Tokyo Kashiwa Japan; ^3^ Artificial Intelligence Research Center, National Institute of Advanced Industrial Science and Technology (AIST) Tokyo Japan; ^4^ Department of Data Science, School of Frontier Engineering Kitasato University Sagamihara Japan

**Keywords:** Hyena architecture, protein language model, protein pre‐training

## Abstract

The emergence of self‐supervised deep language models has revolutionized natural language processing tasks and has recently extended its applications to biological sequence analysis. Traditional language models, primarily based on Transformer architectures, demonstrate substantial effectiveness in various applications. However, these models are inherently constrained by the attention mechanism's quadratic computational complexity, $O\left(L^{2}\right)$, which limits their efficiency and leads to high computational costs. To address these limitations, we introduce ProtHyena, a novel approach that leverages the Hyena operator in protein language modeling. This innovative methodology alternates between subquadratic long convolutions and element‐wise gating operations, which circumvents the constraints imposed by attention mechanisms and reduces computational complexity to subquadratic levels. This enables faster and more memory‐efficient modeling of protein sequences. ProtHyena can achieve state‐of‐the‐art results and comparable performance in 8 downstream tasks, including protein engineering (protein fluorescence and stability prediction), protein property prediction (neuropeptide cleavage, signal peptide, solubility, disorder, gene function prediction), protein structure prediction, with only 1.6 M parameters. The architecture of ProtHyena represents a highly efficient solution for protein language modeling, offering a promising avenue for fast and efficient analysis of protein sequences.

## Highlights

- Novel Architecture: ProtHyena applies Hyena architecture for protein language modeling, outperforming traditional attention‐based models with lower computational costs and fewer parameters.
- State‐of‐the‐Art Performance: Achieves leading results across classification and regression tasks, excelling with diverse protein sequences.
- Efficiency: Optimized for faster training and lower memory usage, enabling scalable modeling of long protein sequences.

## Graphical abstract

The ProtHyena framework is designed for protein sequence prediction and functional analysis, leveraging extensive pre‐training and task‐specific fine‐tuning. During the pre‐training stage, ProtHyena is trained on 14 million sequences from the Pfam database to predict next amino acid residue, using cross‐entropy loss to optimize probabilistic outputs for amino acids. The model's architecture incorporates “Hyena Blocks” that consist of specialized components‐ the Hyena Operator, Feed Forward, and normalization layers—that process sequence embeddings into robust representations. Moving to the fine‐tuning stage, ProtHyena is adapted for diverse downstream tasks including fluorescence prediction, stability assessment, viral protein function analysis, and secondary structure prediction. This targeted fine‐tuning enables ProtHyena to accurately address varied biological tasks, making it a versatile tool for protein function prediction and enhancing applications dependent on accurate protein analysis.

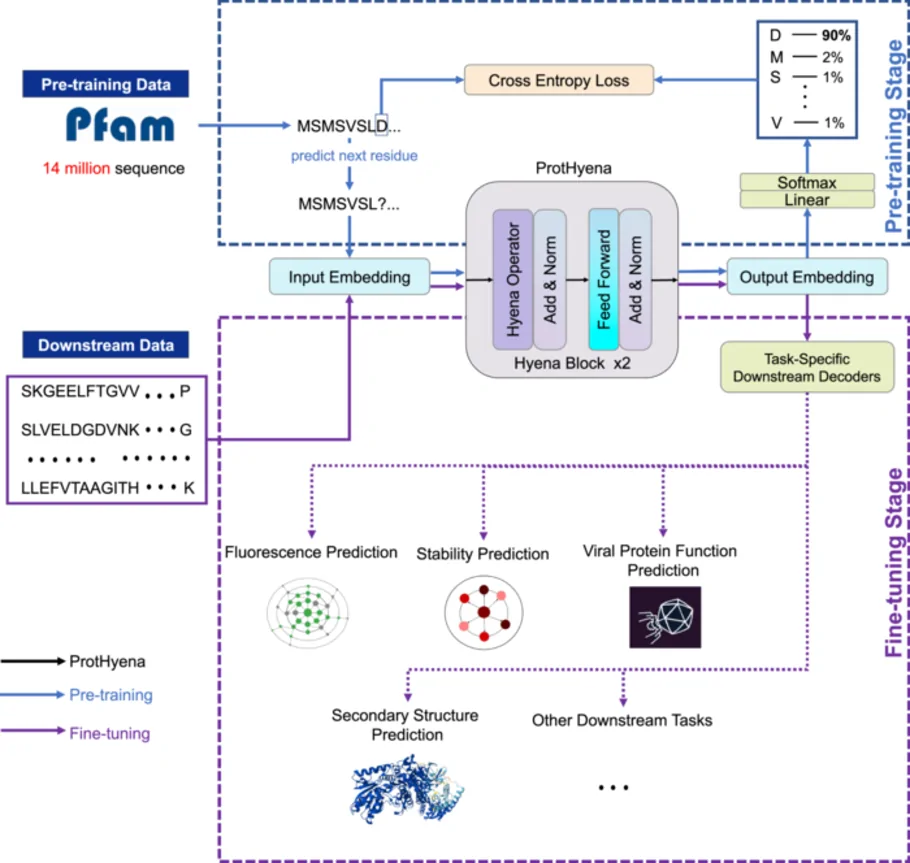

## 1 INTRODUCTION

Proteins, fundamental to the functionality of organisms, play diverse roles in cellular processes, ranging from biochemical reactions catalyzed by enzymes to maintaining cell shape through structural proteins [1]. Understanding protein sequence is crucial in the prediction of protein structures and functions [2]. Recently many supervised machine learning models are developed which enhanced the understanding of protein biology, enabling breakthroughs in protein therapeutics, drug discovery, and synthetic biology [3]. However, despite the exponential growth of protein databases in recent decades, the challenge of obtaining meaningful annotations for these sequences remains a significant barrier [4]. Most protein sequences in these databases lack functional and structural annotations, highlighting the necessity for efficient analysis methods that can capitalize on the wealth of unlabeled protein sequences.

Protein language models (PLMs) adopt self‐supervised pre‐training approaches from natural language processing (NLP), such as ESM2 [5], ProtBert [6] and ProtT5 [6] has revolutionized the representation learning of protein sequences. This methodology involves pre‐training large language models on millions of unlabeled protein sequences to learn universal embeddings, which are then fine‐tuned for various specific protein tasks. The success of these models, particularly due to the attention mechanism, hinges on their ability to scale and facilitate in‐context learning, enabling generalization to unseen data and tasks [7]. However, a significant limitation of these models is the quadratic computational cost associated with the length of input sequences. This cost constraint severely limits the contextual capacity of the models and hinders their application to longer sequences [8].

In addressing the computational demands of Transformer‐based models, various strategies have been employed, such as linearized, low‐rank, and sparse approximations [9, 10, 11] to reduce the computational complexity of self‐attention. Although these methods effectively reduce the computational cost, they often introduce a compromise between model expressivity and processing speed. However, it is necessary to integrate these approximations with standard attention layers to achieve a performance comparable to that of conventional Transformer‐based model, thereby creating a hybrid approach that balances efficiency and effectiveness. Protein language foundation models (PLFMs) like Protein RoBERTa [12] have pioneered the use of Longformer [13] to process up to 2,048 tokens for protein sequence inputs. Similarly, scBERT [14] leverages the Performer [15] for analyzing large single‐cell gene expression data, and MolFormer [16] incorporates linear attention [17] to more effectively capture spatial relations between chemical atoms.

To match the high performance of Transformers with a lower computational burden, it is crucial to obtain operators that maintain attention's three defining properties: data control, sublinear parameter scaling, and unrestricted context. The recent development of Hyena [18] successfully achieves this by introducing an operator characterized by a recurrent structure, which combines two subquadratic primitives: a long convolution and an element‐wise multiplicative gating. This innovative operator retains the core attributes of attention, ensuring efficient processing across lengthy sequences without compromising on the ability to model complex dependencies [18]. Motivated by these advancements, we develop ProtHyena which pre‐training a protein language model by incorporating the Hyena operator. This architecture can unlock the potential to capture both the long‐range and single amino acid resolution of protein sequences over attention‐based approaches much more efficient. The pre‐trained model can also be fine‐tuned to apply on eight diverse downstream tasks. We examine the performance of our model and found our model approaches or even surpasses state‐of‐the‐art performance in many downstream tasks with a minimal number of parameters. In all experiments, we observe that our model significantly enhances task performance compared to similarly configured attention‐based models. This improvement underscores the substantial value of incorporating the Hyena architecture.

## 2 RESULTS

### Overview of ProtHyena in protein language modeling

**Figure 1:**
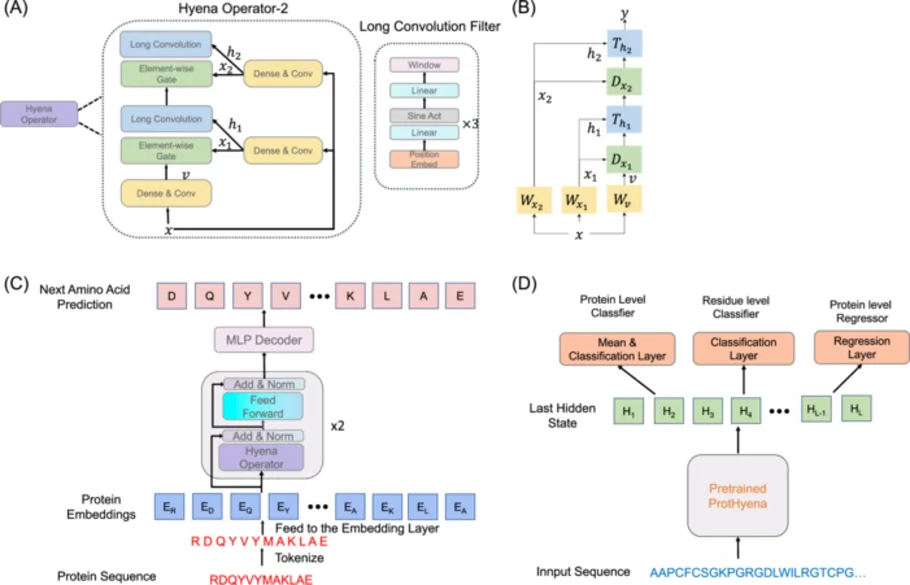
Overview of our proposed framework for ProtHyena. (A) ProtHyena utilizes Hyena operator as its core architecture. The Hyena Operator features long convolutions and element‐wise gating layers, which process the input after modification by dense layers and short convolutions. (B) Illustration of a simple order = 2 Hyena operator. The input protein sequence, x, is projected through three different projection matrices to yield sequences v, $x_{1}$, and $x_{2}$. Sequence v is subjected to alternating gating operations controlled by a diagonal matrix D, and long convolution operations controlled by a Toeplitz matrix composed of h. (C) The Generative Pre‐training strategy. The input protein sequence is tokenized to amino acids. The model structure includes two layers of Hyena Blocks; each layer consists of a Hyena Operator, a fully connected network, residual connections and layer normalization. The embeddings from the last layer are decoded into amino acids using a Multi‐layer Perceptron (MLP). (D) The fine‐tuning process applied to the pre‐trained model across eight downstream tasks.

In this study, we develop a fast and parameter‐efficient protein language foundation model (ProtHyena) that incorporates the Hyena operator (Figure 1A, B) for protein sequence analysis. Hyena operator incorporating long convolutions and element‐wise gating and a fast fourier transform (FFT) convolution method is used for fast and efficient processing (Figure 1A). ProtHyena applies a generative pre‐trained Transformer strategy, substituting its attention layer with the Hyena operator and employing an autoregressive task for pre‐training (Figure 1C) using 14 million protein sequences from the Pfam data set [19], We then fine‐tuned our pre‐trained ProtHyena on various protein‐related tasks (Figure 1D). To compare with other published models, we deliberately choose 8 benchmarks which reflects the broad spectrum of protein research, encompassing aspects of protein engineering, protein property and functions prediction, protein structure understanding tasks [20].

### Enhanced efficiency and performance of ProtHyena in protein language modeling

**Figure 2:**
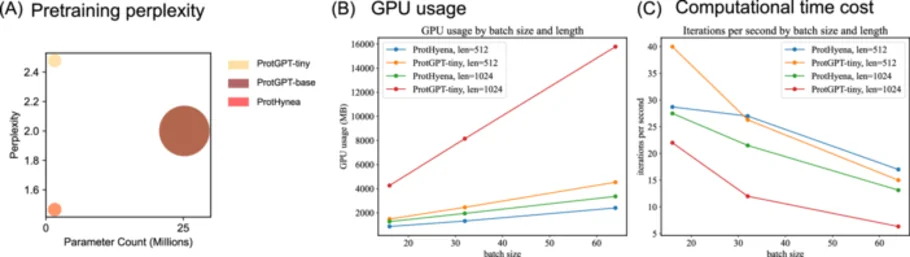
Comparison of parameters, performance, and resource usage between various models. (A) Comparison of parameters and pretraining between ProtGPT‐tiny, ProtGPT‐base and ProtHyena. Lower perplexity indicates better performance in predicting protein sequences. (B) GPU memory consumption across different models when operated under various batch sizes and maximum sequence lengths. (C) Computational time cost (the number of iterations per second) for different models under varying batch sizes and sequence lengths.

To evaluate the effectiveness of ProtHyena based on the Hyena operator compared to attention‐based protein language models, we also pretrain two decoder‐only attention‐based models: ProtGPT‐tiny, with a parameter size of 1.6 million, matching that of ProtHyena, and the larger ProtGPT‐base, encompassing 25.2 million parameters (Figure 2A). Both models are trained on the same 14 million protein sequence in Pfam data set as ProtHyena, using a batch size of 256 and for an equal number of training steps. The detailed parameter comparison among the other different protein language models used in this study is presented in Figure S1.

Our findings show that ProtHyena achieved the lowest perplexity among the models, which indicates better predictive accuracy (Figure 2A). In language modeling, perplexity is a metric used to assess how well a model predicts the next element in a sequence, such as amino acids in proteins. A lower perplexity means the model is less uncertain about its prediction. For example, if a model has a perplexity of 20, it is effectively choosing between 20 possible next elements in the sequence. Theoretically, the best possible perplexity is 1, meaning the model is completely certain about predicting the next amino acid.

In our study, ProtHyena achieves a significantly lower perplexity compared to the other models, with values of 2.48 for ProtGPT‐tiny, 2.00 for ProtGPT‐base, and 1.47 for ProtHyena. This means that, on average, ProtGPT‐tiny considers 2.48 options, ProtGPT‐base considers 2.00 options, and ProtHyena considers only 1.47 options when predicting the next amino acid. ProtHyena's lower perplexity, even compared to the larger ProtGPT‐base, highlights its confidence and accuracy in predicting amino acids, demonstrating the model's efficiency and the effectiveness of the Hyena operator in handling the complexities of protein sequences.

To further validate the efficiency of ProtHyena compared to Transformer‐based models, we also conduct tests on an NVIDIA RTX 3090 GPU. We compare the memory usage and computational time cost of ProtHyena and ProtGPT‐tiny, both having the same number of parameters (1.6 million), across various batch sizes (16, 32, 64) and maximum sequence lengths (512, 1024). We choose ProtGPT‐tiny as a control model to ensure a fair comparison of runtime and GPU memory usage, given that it has the same number of parameters as ProtHyena. For models with a larger number of parameters, the runtime and GPU usage are understandably higher, so comparing these directly would not provide an accurate assessment of ProtHyena's efficiency.

The results (Figure 2B) show that ProtHyena consistently uses significantly less memory than ProtGPT‐tiny. Notably, the increase in memory usage when doubling the sequence length from 512 to 1024 is not substantial for ProtHyena. In terms of computational time cost, measured as the number of forward iterations per second, ProtGPT‐tiny shows a slight advantage only in a limited scenario with a smaller data volume: a batch size of 16 and a sequence length of 512 (Figure 2C). However, in practical pre‐training stage, where larger datasets and batch sizes are typically used, the efficiency of ProtHyena improves significantly. Under all other tested conditions, especially with larger batch sizes (64) and longer sequence lengths (1024), ProtHyena is approximately twice as fast as ProtGPT‐tiny. These findings indicate that while ProtGPT‐tiny may have a slight speed advantage in specific, small‐scale scenarios, ProtHyena is more resource‐efficient and faster for large‐scale pre‐training. We also provide the average training time for one epoch across six downstream tasks in Supplementary Table S1, further confirming that ProtHyena is not only faster during pre‐training but also during fine‐tuning.

### ProtHynea can accurately predict fluorescence landscape

**Figure 3:**
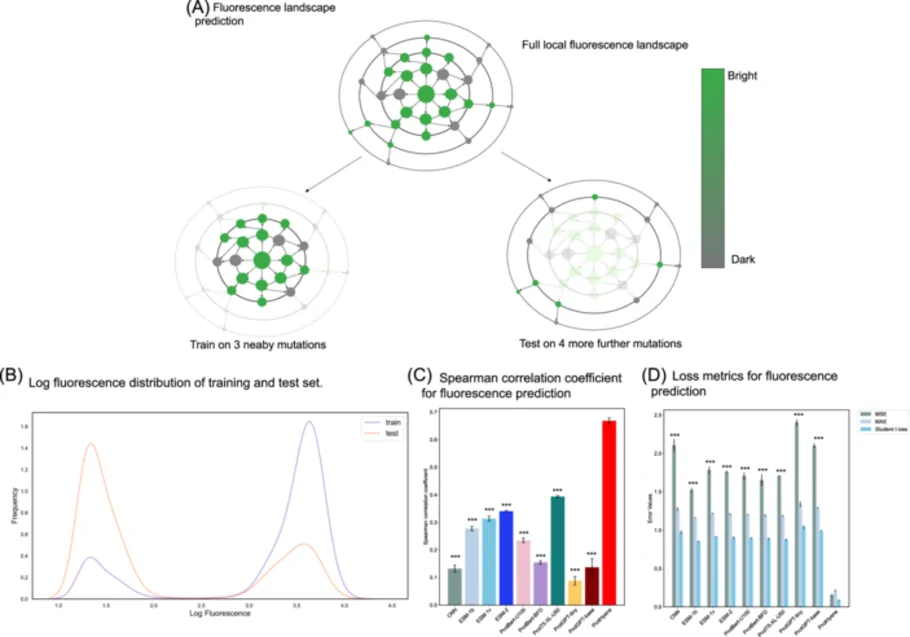
Fluorescence landscape prediction task. (A) The wild type avGFP (center) and most single mutants (innermost circle) emit green fluorescence. Genotypes with multiple mutations can demonstrate negative epistasis, where combinations of neutral mutations result in nonfluorescent (grey) phenotypes, or positive epistasis, where a mutation in a nonfluorescent genotype restores fluorescence. Training set is 3 mutations which are relative brighter than 4 more further mutations test set. (B) Log fluorescence distribution of training and test set. The training set is centered around 3.6, while the test set is centered around 1.4. (C) Spearman correlation coefficient of ProtHyena and other protein language models for fluorescence landscape prediction. (D) Loss metrics of ProtHyena and other protein language models for fluorescence landscape prediction. Loss metrics we used include MSE, MAE and Student t loss. Statistical significance, determined by Student's t‐test performed between other models and ProtHyena, is indicated as follows: *p < 0.05, **p < 0.01, ***p < 0.001.

*Aequorea victoria*'s green fluorescent protein (avGFP) is a widely used marker in molecular and cellular biology due to its ability to fluoresce naturally, making it invaluable for visualizing and tracking biological processes in real time [21]. Our investigation begins with a task predicting the protein fluorescence landscape of *Aequorea victoria*'s green fluorescent protein (avGFP), using data derived from tens of thousands of avGFP genotypes subjected to random mutagenesis [21]. This data shows that the wild‐type and most single‐mutant genotypes of avGFP exhibit bright green fluorescence. However, as more mutations accumulate, some genotypes may experience negative epistasis, leading to a loss of fluorescence (grey phenotypes). In other cases, positive epistasis can occur, restoring fluorescence even in nonfluorescent genotypes (Figure 3A). By evaluating fluorescence levels across a wide range of genetically modified avGFP sequences, we can better understand how mutations affect protein fluorescence. This method not only reveals the influence of genetic variations on protein stability but also helps in designing proteins with desired traits and provides insight into evolutionary dynamics in similar proteins [22].

**Figure 4:**
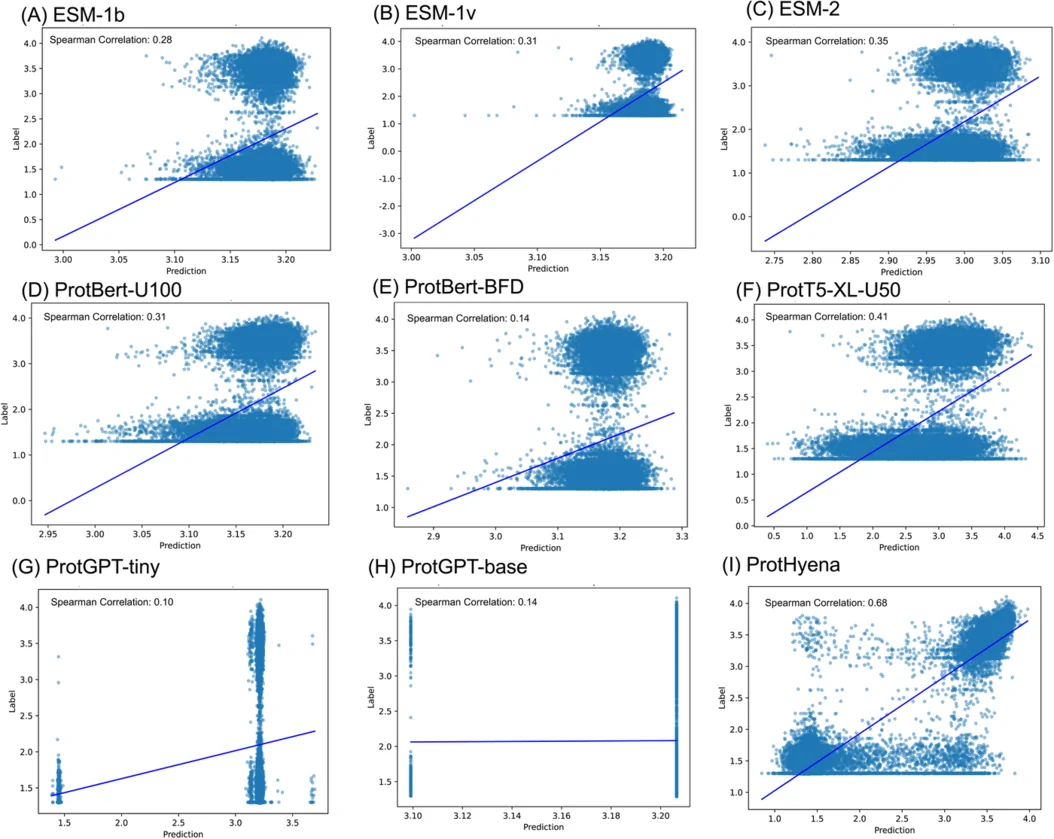
Scatter plots with fitting lines on fluorescence task. The plots compare predicted values (x‐axis) and true labels (y‐axis) across different models. Each plot shows a linear fit line and the Spearman correlation coefficient, indicating prediction accuracy. (A) ESM‐1b, (B) ESM‐1v, (C) ESM‐2, (D) ProtBert‐U100, (E) ProtBert‐BFD, (F) ProtT5‐XL‐U50, (G) ProtGPT‐tiny, (H) ProtGPT‐base, (I) ProtHyena.

For training, we use a data set consisting of variants within a Hamming distance of 3 from the parent GFPs, which predominantly display brighter phenotypes due to fewer mutations. In contrast, the test set includes variants with four or more mutations, typically resulting in darker phenotypes (Figure 3B). By training on proteins with fewer mutations (three or fewer) and testing on proteins with more mutations (four or more), we effectively evaluate the model's generalizability, demonstrating how well it performs on unseen data. To compare ProtHyena, we evaluate one traditional deep learning model, a convolutional neural network (CNN), alongside several pre‐trained protein language models (ESM1‐b, ESM‐1v, ESM‐2b, ProtBert‐U100, ProtBert‐BFD, ProtT5‐XL‐U50), as well as two protein language models that we pretrain, ProtGPT‐tiny and ProtGPT‐base. Despite their large number of parameters and training on extensive datasets, CNN and other pre‐trained protein language models struggle to capture the subtle differences in fluorescence intensities caused by similar mutations. In contrast, ProtHyena achieves a Spearman correlation coefficient of 0.68, surpassing the next best model, ProtT5‐XL‐U50, by 0.2 (Figure 3C). Additionally, ProtHyena significantly outperforms other models in terms of mean squared error (MSE), mean absolute error (MAE), and Student's *t*‐loss, with respective scores of 0.15, 0.21, and 0.09 (Figure 3D). In the scatter plots with fitting lines (Figure 4) for various protein language models, while most models tend to overpredict for lower true values (around 1.5 to 2.0), ProtHyena performs best at capturing both clusters of data points—those with lower values (around 1.5 to 2.0) and higher values (around 3.0 to 4.0). ProtHyena has the highest concentration of predicted points near both low and high true values, reflecting its superior spearman correlation score.

### ProtHyena enhance the prediction of protein stability

Protein stability is crucial for the proper function and longevity of proteins within biological systems [23]. Stable proteins maintain their structural integrity under physiological conditions, ensuring that they can perform their intended biological activities effectively [24]. To further test the capabilities of ProtHyena, we evaluate its performance on stability landscape prediction task.

**Figure 5:**
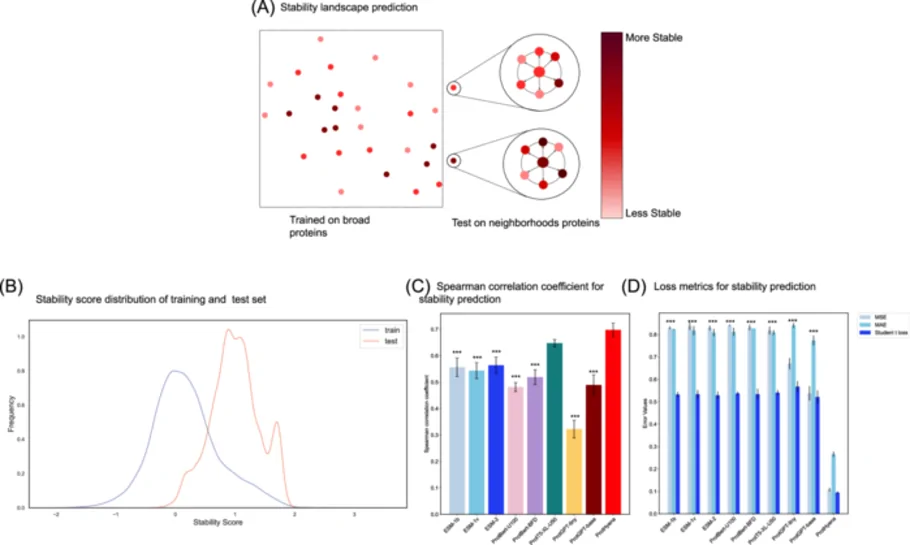
Stability landscape prediction task. (A) The Stability task involves training on a wide array of proteins and then testing on proteins that are one mutation away from the most promising candidates identified in the sample. (B) Stability score distribution of training and test set. (C) Spearman correlation coefficient of ProtHyena and other protein language models for stability landscape prediction. (D) Loss metrics of ProtHyena and other protein language models for stability landscape prediction. Loss metrics we used include MSE, MAE and Student *t* loss. Statistical significance, determined by Student's *t*‐test performed between other models and ProtHyena, is indicated as follows: *p < 0.05, **p < 0.01, ***p < 0.001.

We collect data from [25], which includes 53,679 sequences for training, 2,447 for validation, and 12,839 for testing. Each protein sequence has a real‐value label indicating the most extreme conditions under which the protein maintains its fold above a concentration threshold (a proxy for intrinsic stability). This data set is also suitable for testing generalizability to unseen proteins. The training data set includes proteins from four iterative rounds of experimental mutations design, while the testing data set consists of proteins that are single mutation neighbors to the most promising candidates (Figure 5A). This arrangement results in a distribution shift in the stability scores between the training and testing datasets (Figure 5B). The stability score refers to the free energy of unfolding ($\Delta G_{u\mathrm{nf}}$) measured at 40°C through thermal denaturation. Positive values indicate that the protein's folded state is more stable, meaning it requires energy to unfold. In contrast, negative values suggest that the unfolded state is more stable, indicating the protein is more likely to unfold under these conditions.

**Figure 6:**
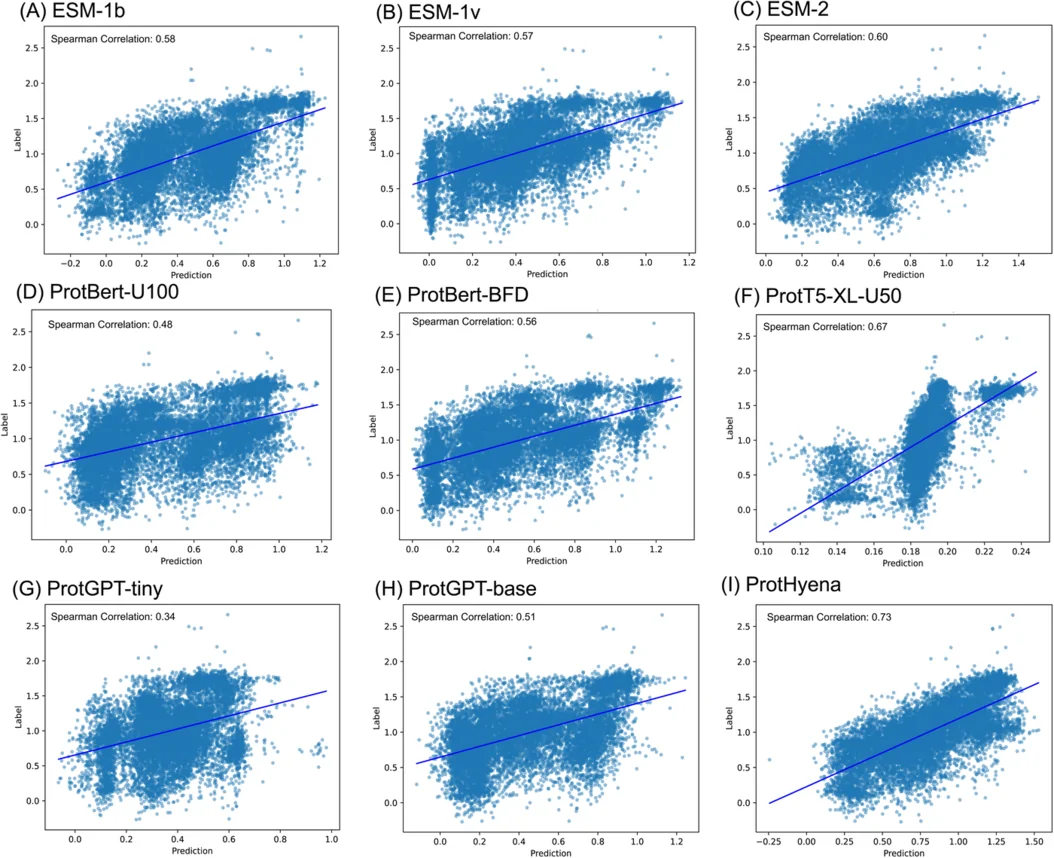
Scatter plots with fitting lines on stability task. The plots compare predicted values (x‐axis) and true labels (y‐axis) across different models. Each plot shows a linear fit line and the Spearman correlation coefficient, indicating prediction accuracy. (A) ESM‐1b, (B) ESM‐1v, (C) ESM‐2, (D) ProtBert‐U100, (E) ProtBert‐BFD, (F) ProtT5‐XL‐U50, (G) ProtGPT‐tiny, (H) ProtGPT‐base, (I) ProtHyena.

In this benchmark, ProtHyena achieves a Spearman correlation coefficient of 0.70. We conducted a Student's *t*‐test comparing ProtHyena with other models. Except for ProtT5‐XL‐U50, ProtHyena significantly outperform all other models (Figure 5C). Additionally, ProtHyena shows superior accuracy in its predictions, with much lower mean squared error (MSE), mean absolute error (MAE), and Student's *t*‐loss scores—0.10, 0.26, and 0.09, respectively (Figure 5D). While the correlation between ProtT5‐XL‐U50 and ProtHyena in predicting stability scores is similar, ProtT5‐XL‐U50 shows higher MSE and MAE. This is because ProtT5‐XL‐U50 tends to predict lower stability scores than the true values. This could be due to the Transformer‐based architecture of ProtT5‐XL‐U50, which relies on a self‐attention mechanism. This mechanism is highly effective at capturing long‐range dependencies and global information in sequences, enabling the model to identify distant interactions between amino acids. As a result, the model excels at capturing relative relationships between samples, explaining its high Spearman correlation. In other words, it can differentiate between more stable and less stable protein sequences. However, this focus on global patterns can sometimes cause Transformers to miss subtle local features. In protein stability tasks, small changes in local amino acid structures can have a significant impact on stability. Since Transformers prioritize global sequence characteristics, they may not accurately predict these fine‐grained stability changes, leading to discrepancies in predicted values. Figure 6 shows scatter plots with fitting lines, where we can see that ProtHyena's predictions are more densely clustered, indicating a higher correlation. These findings suggest that ProtHyena's predictions are significantly more precise, more robust to anomalies, and less affected by experimental noise commonly found in biological datasets.

### ProtHyena predicts protein property accurately

Predicting protein properties is crucial, as it offers vital insights into the behaviors and functions of proteins. In this part, we focus on four key tasks related to the prediction of protein properties and functions: Neuropeptide Cleavage Prediction [26] involves predicting the sites where larger precursor proteins are enzymatically cleaved to produce smaller peptides. These peptides function in neurotransmission and various physiological processes, indicating their significance in neural and bodily functions. In Protein Disorder Prediction task [27], protein sequence and classify each residue as either “ordered” or “disordered”. Solubility Prediction [28], aims to predict whether a given protein is soluble or not. Signal Peptide Prediction [29], predicting whether an entire protein sequence from four different species groups—Eukarya, Archaea, Gram‐positive bacteria, and Gram‐negative bacteria has a signal peptide or not. These tasks are crucial for understanding the biological roles and behaviors of proteins under various conditions.

In Figure S2, we present the label distribution for the four binary classification tasks. Notably, only the solubility task has relatively balanced labels. In the Neuropeptide Cleavage Prediction task, the ratio of 0 to 1 is approximately 3:7. For Disorder Prediction, almost all labels are 1, with very few 0 s. In the Signal Peptide Prediction task, the ratio of 0 to 1 is about 2:8. To better evaluate model performance on these imbalanced datasets, we use metrics such as macro F1, Matthew's Correlation Coefficient (MCC), and Area Under the Receiver Operating Characteristic (AUROC).

**Figure 7:**
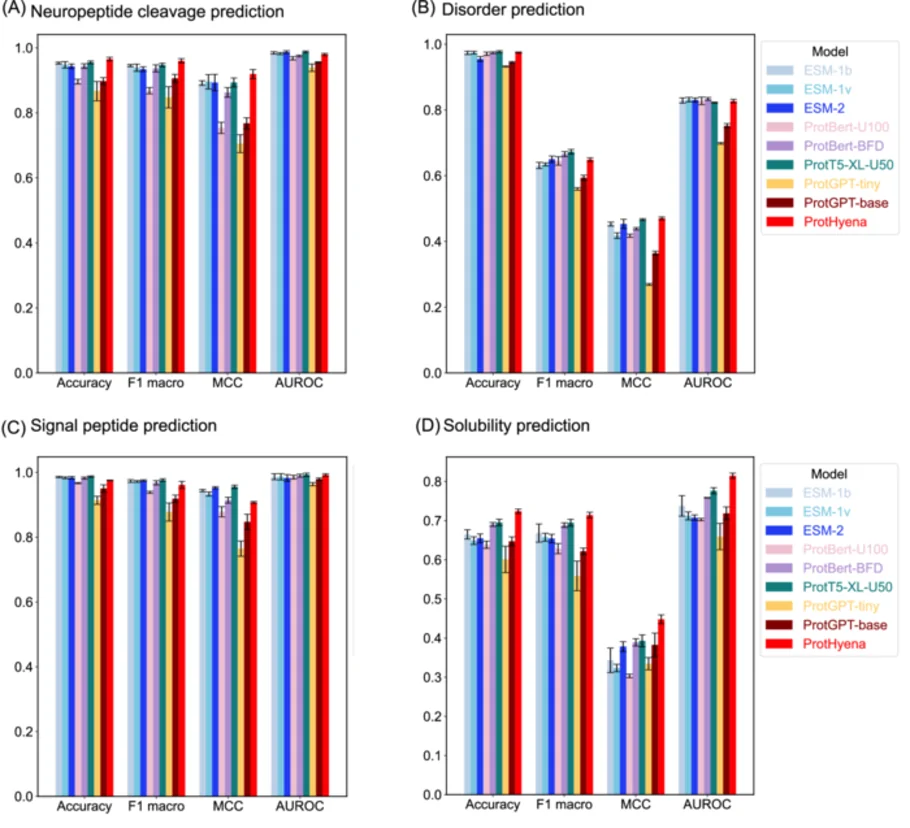
Evaluation on different protein property tasks. Accuracy, F1 macro score, Matthew's Correlation Coefficient (MCC) and Area Under the Receiver Operating Characteristic Curve (AUROC) for score were used for each task. (A) Evaluation of various models for neuropeptide cleavage prediction. (B) Evaluation of various models for disorder prediction task. (C) Evaluation of various models for signal peptide prediction. (D) Evaluation of various models for solubility prediction.

In evaluating model performance, ProtHyena, our developed model, shows outstanding results. In Neuropeptide Cleavage Prediction, ProtHyena leads with an accuracy of 0.9656 and a MCC of 0.9194, demonstrating its superior capability to predict crucial cleavage sites accurately compared to other leading models such as ESM and ProtBert series (Figure 7A). For Protein Disorder Prediction, ProtHyena also excels, achieving the highest MCC of 0.4703 among the models and a competitive F1 macro score of 0.3276, suggesting its effectiveness in handling complex protein characteristics (Figure 7B).

In Signal Peptide Prediction, although slightly behind ProtT5‐XL‐U50 in F1 and MCC, ProtHyena demonstrates robust accuracy at 0.9754 and an impressive AUROC of 0.9921, highlighting its capability in accurately detecting signal peptides across different species groups (Figure 7C). Finally, in Solubility Prediction, ProtHyena outperforms all models with the highest accuracy of 0.724 and AUROC of 0.8146, confirming its strong predictive power in determining protein solubility, which is often challenging to predict accurately (Figure 7D).

**Figure 8:**
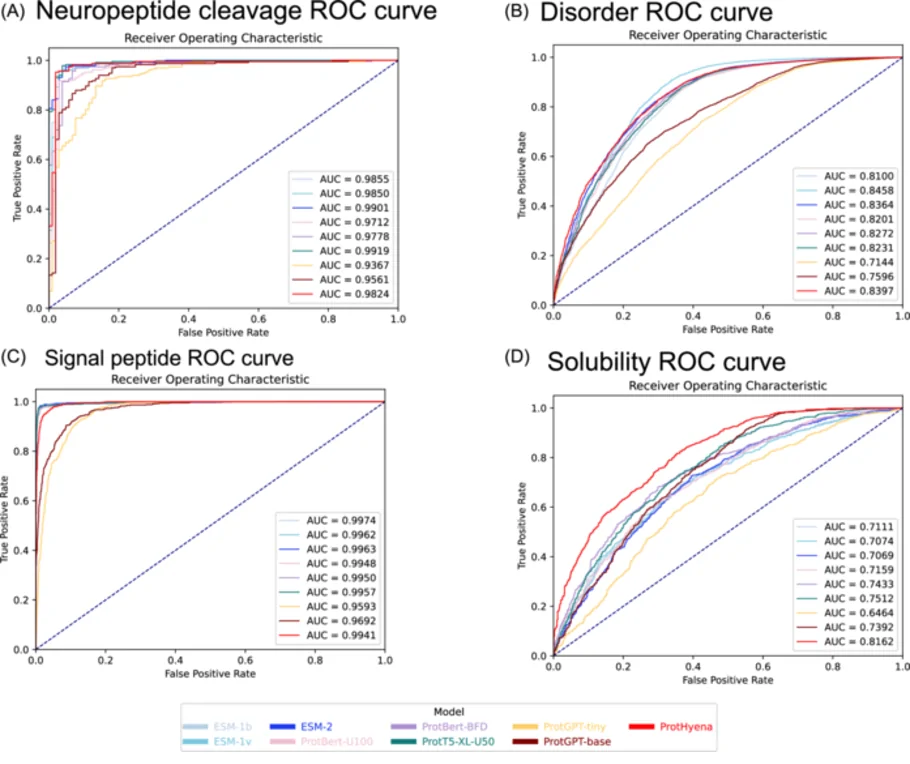
Receiver operating characteristic (ROC) curves on different protein property tasks. The ROC curves illustrate the classification performance of different models across four protein property prediction tasks: (A) Neuropeptide cleavage, (B) Disorder, (C) Signal peptide, and (D) Solubility. The x‐axis represents the false positive rate, and the y‐axis represents the true positive rate. Each curve corresponds to a model, with the AUC (area under the curve) values indicating the model's ability to distinguish between classes for each task.

We plot the receiver operating characteristic (ROC) curves for all 9 protein language models to provide clearer comparisons in Figure 8. In the three imbalanced tasks (Neuropeptide Cleavage, Disorder, and Signal Peptide), the models performed similarly, with no significant differences in their ability to handle imbalanced data. However, in the balanced task (Solubility), ProtHyena shows the best performance, highlighting its superior capability in balanced datasets. This suggests that while other models may struggle to capture subtle distinctions in balanced settings, ProtHyena excels due to its ability to effectively handle both local and global features, giving it an edge over the other models.

### Gene function prediction of viral proteins using ProtHyena

Gene function prediction is pivotal for understanding gene roles and interactions within biological systems, particularly in fields such as microbial ecology and virology [30]. These predictions are especially valuable for identifying the functions of newly discovered genes in environmental samples, where many sequences lack known references [31]. Protein language models (PLMs) enhance this field by capturing the physico‐chemical properties and functional homology of amino acids directly from sequence data [32, 33]. PLMs transcend traditional sequence homology methods, which often fail due to the rapid evolutionary changes in proteins, thus facilitating more accurate annotation of viral protein families [34]. This improved annotation significantly advances our understanding of viral functions and supports biological discoveries, including the identification of key viral enzymes and structural proteins in various ecological samples [35].

**Figure 9:**
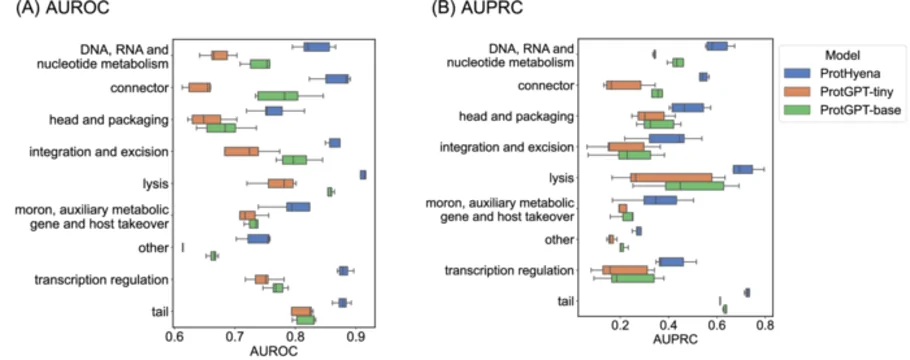
Functional category classification of PHROG viral protein families. (A) Area under the receiver operating characteristic curve (AUROC) were used for evaluation of ProtGPT‐tiny, ProtGPT‐base, and ProtHyena. (B) Area under the precision‐recall curve (AUPRC) were used for evaluation of ProtGPT‐tiny, ProtGPT‐base, and ProtHyena.

To further assess the effectiveness of ProtHyena in gene function prediction, we apply our model in viral gene function annotation. Our annotations are based on the Prokaryotic Virus Remote Homologous Groups (PHROGs) database [36], a meticulously curated collection designed to capture remote sequence homology and manually annotated into high‐level functional categories. PHROGs version3 contains 868,340 protein sequences grouped into 38,880 families. Of these families, 5,088 are classified into nine functional classes. After removing proteins without annotations, we obtain 472,683 viral proteins for further analysis. Utilizing a five‐fold cross‐validation approach, as described in [37], we compare the performance of ProtHyena with other models such as ProtGPT‐tiny and ProtGPT‐base. Our results indicate that ProtHyena outperforms the other models, achieving an average area under the receiver operating characteristic curve (AUROC) of 0.84 (Figure 9A) and an average area under the precision–recall curve (AUPRC) of 0.50 across all classes and folds (Figure 9B), demonstrating its capability in viral gene function prediction.

## 3 DISCUSSION

Protein language models (PLMs) have revolutionized the field of bioinformatics by enabling advanced analysis and prediction of protein structures and functions [38]. These models, inspired by natural language processing, treat amino acid sequences as “sentences” and leverage deep learning techniques to understand and predict protein properties [39]. However, recent developed protein language models encounter computational challenges. In this study, we present ProtHyena, a novel protein language model that integrates the Hyena operator to address the computational challenges encountered by attention‐based protein language models. ProtHyena not only efficiently processes protein sequences using less GPU memory and computational time cost but also matches, and sometimes outperform, state‐of‐the‐art performance in a wide array of protein‐related downstream tasks. With only a fraction of the parameters required by traditional models, ProtHyena exemplifies a significant leap forward in the field of protein sequence analysis.

Our comprehensive pre‐training and fine‐tuning on the expansive Pfam data set across 8 different tasks have demonstrated the model's ability to capture complex biological information with high accuracy. In five of these tasks, our model achieves state‐of‐the‐art performance, while in the remaining tasks, it demonstrates performance comparable to existing benchmarks. The adoption of the Hyena operator enables ProtHyena to perform at a subquadratic time complexity, making it a highly fast and efficient tool for analyzing biological sequences.

The importance of protein design lies in its ability to revolutionize both industry and academia by enabling the creation of proteins with user‐defined functionality, which can lead to breakthroughs in medicine and biotechnology. The performance of ProtHyena in fluorescence landscape and stability prediction holds promise for applications in protein design and engineering, offering insights into mutation impacts to facilitate the creation of proteins with tailored properties. Furthermore, its superior performance can contribute to a deeper understanding of molecular evolution, shedding light on the development of fluorescence and other protein functionalities. This knowledge is not only pertinent to evolutionary studies and population genetics but also has practical implications for designing fluorescent markers and reporters critical in gene expression analysis, protein localization, and interaction studies—key elements in both fundamental research and therapeutic development.

In viral gene function prediction task, ProtHyena exhibits better performance with other models. This makes it a powerful tool for discovery and an excellent complementary method to traditional alignment‐based approaches. These capabilities are pivotal for advancing our understanding of the functional dynamics of viral populations globally.

Our comparative evaluation on different protein property and functions tasks reveals that models based on the ESM, ProtBert or ProtT5 frameworks perform similarly to ProtHyena across various tasks. Specifically, ProtHyena demonstrates state‐of‐the‐art results in relatively simpler tasks such as Neuropeptide cleavage prediction and Disorder prediction. This is attributed to ProtHyena's effective pretraining on small‐scale datasets with fewer parameters. Conversely, for transformer‐based models, increasing the model size and training scale, as seen with ESM‐2, ProtBert, and ProtT5, enhances performance but reaches a plateau, indicating a limit to performance gains from scaling alone. These results underscore the robust and stable performance of ProtHyena across multiple tasks highlights its effectiveness and cross‐task applicability.

In predicting protein secondary structures, ProtHyena exhibits suboptimal performance compared to ESM series [40, 41], ProtBert and ProtT5‐XL [6] models (Figure S3). Protein secondary structure prediction, being a residue‐level task, involves a vast potential space of possibilities $3^{L}$ for a protein of length L, considering three structural categories per residue. In contrast, protein disorder prediction, also a residue‐level task but binary, involves a significantly smaller search space of $2^{L}$. The exponential complexity of secondary structure prediction underscores the necessity for extensive data‐driven learning and suggests that comprehensive learning of each residue's role through self‐attention mechanisms is crucial. A hybrid model that combines convolutional layers with multi‐head attention mechanisms and rotary position embeddings, as introduced by Evo [42], may enhance performance in predicting protein secondary structures.

In the future, we aim to scale up ProtHyena to fully leverage its capabilities and precision, while also exploring masked language modeling methods for pre‐training to broaden its applicability. The architecture we have introduced sets a new precedent for protein language models, offering a promising framework for future advancements in the field.

## 4 CONCLUSION

In this paper, we develop ProtHyena, a new protein language model based on the Hyena architecture. ProtHyena overcomes the challenges of traditional attention‐based models by significantly lowering memory requirements and training time, while still achieving high accuracy in various protein‐related tasks. Its strong performance in modeling protein sequences, especially in predicting fluorescence and stability landscapes, highlights its potential for practical applications in protein design and the prediction of protein functions and properties.

In conclusion, ProtHyena's successful integration of cutting‐edge architecture with biological tasks not only showcases its practical application but also provides a solid foundation for future innovations in protein analysis and design.

## 5 METHODS

### Protein sequence data for pre‐training and data for downstream tasks

Consistent with prior studies [25, 43], we collect 14 million protein sequences across diverse evolutionary taxonomy from Pfam database (release version 32.0, accessed May 16, 2024) for pre‐training [44].

The following are the datasets for the downstream tasks, 54,025 *Aequorea victoria*'s green fluorescent protein are downloaded from TAPE data set [25]. The training sets are in 3 mutations neighborhood of the original protein, while test data is a sample from the 4–15 mutations neighborhood. The training set contains 21,446 sequences, the validation set includes 5,362 sequences, and the test set comprises 27,217 sequences.

We obtain the data set consisting of 56,126 training sequences and 12,851 test sequences from [25] originates in [45] for stability prediction task. The training set is derived from four rounds of experimental data measuring the stability of numerous candidate proteins. The test set comprises eighteen single mutation neighborhoods around promising proteins identified during these four rounds of experimentation.

We obtain the neuropeptide cleavage benchmark consisting of 3,366 proteins derived from an earlier work [46]. The goal is to predict if a basic residue (K or R) undergoes cleavage, where all candidate sequences have a signal peptide.

We obtain 11361 proteins from [20] for protein disorder prediction task. The goal is to predict each residue in the sequence either “disordered” or “not”.

We obtain a data set consisting of 28,972 soluble and 40,448 insoluble protein sequences for the solubility prediction task from [28]. An independent test set, comprising 1,000 soluble and 1,001 insoluble protein sequences, is derived from [47].

We obtain the signal peptide benchmark consisting of 24,910 protein sequences, aimed at predicting whether an entire protein sequence has a signal peptide or not, derived from SignalP 5.0 [29].

We obtain 472,683 viral protein sequences from PHROGs v3 [36], which are clustered into 5,088 families. These families are annotated across nine functional classes for viral protein function prediction.

We obtain 13474 proteins for secondary structure prediction at the residue level, out of 3 classes (Helix, Strand and Other) from TAPE benchmark [25].

We summarize the downstream tasks in Table 1.

**Table 1:** Summary of downstream fine‐tuning tasks. For multiclass classification tasks, the number of classes is in the parentheses.

Task	Type	Resolution	Metric	Source
Secondary structure	Multi‐class(3)	Residue	Accuracy, F1macro, AUROC	[25, 27]
Gene function prediction	Multi‐class(9)	Protein	AUROC, AUPRC	[36, 37]
Neuropeptide cleavage	Binary	Protein	Accuracy, F1macro, MCC, AUROC	[26]
Disorder	Binary	Residue	Accuracy, F1macro, MCC, AUROC	[27]
Signal peptide	Binary	Protein	Accuracy, F1macro, MCC, AUROC	[29]
Solubility	Binary	Protein	Accuracy, F1macro, MCC, AUROC	[28]
Fluorescence	Regression	Protein	Spearman correlation coefficient, MSE, MAE, Student t loss	[21]
Stability	Regression	Protein	Spearman correlation coefficient, MSE, MAE, Student t loss	[25, 45]

### Tokenization

In our study, we use the natural protein vocabulary, treating each amino acid as an individual token. These tokens represent the 20 standard amino acids, denoted by the characters ‘D’, ‘N’, ‘E’, ‘K’, ‘V’, ‘Y’, ‘A’, ‘Q’, ‘M’, ‘I’, ‘T’, ‘L’, ‘R’, ‘F’, ‘G’, ‘C’, ‘S’, ‘P’, ‘H’, and ‘W’. Additionally, we include five characters designated for less common amino acids and special character tokens to represent padding, separation, and unknown characters. Each of these tokens is mapped to an embedding with dimension D, facilitating the representation and processing of protein sequences within our model framework. This method allows for a comprehensive and accurate encoding of protein sequences, crucial for effective analysis and modeling.

### ProtHyena architecture

The overall framework of our ProtHyena framework is shown in Figure 1A. The Hyena operator is defined by a recurrent structure, incorporating long convolutions and element‐wise gating, as shown in the equation:

$$y=x_{N}\cdot \left(h_{N}*\left(x_{N-1}\cdot \left(h_{N-1}*\left(\ldots x_{1}\cdot \left(h_{1}*v\right)\right)\right)\right)\right)$$

where $v,x_{1},\ldots ,x_{N}$ denote the input projections, N represents the number of recurrences, ⋅ indicates the element‐wise gating and *denotes the long convolution.

To be more specific, Figure 1B provides a visual representation of the Hyena operator with N=2. When processing an input sequence x of length L, the Hyena operator is applied as follows:

$$y=H(x_{1},x_{2})v$$

$$H(x_{1},x_{2})=D_{x_{2}}T_{h_{2}}D_{x_{1}}T_{h_{1}}$$

where $\left(x_{1},x_{2},v\right)$ are projections of the inputs, specifically generated by a linear projection followed by 1‐dimension short convolution. The Toeplitz matrix $T_{h_{k}}\in R^{L\times L}$ is created from a learnable long convolution filter generated as the output of a neural network, with each element ${\left(T_{h_{k}}\right)}_{\mathrm{ij}}=h_{i-j}$. The values of the convolution filter are derived from a small neural network, denoted as γ(θ), which takes the time index and protein embeddings as its input. This function, expressed as $h_{t}=\gamma (\theta (t))$, allows the operator to efficiently handle sequences without a linearly increasing in the number of parameters. This setup ensures that our model remains parameter‐efficient even as it scales to accommodate very long sequences.

Moreover, the diagonal matrices $D_{x_{1}},D_{x_{2}}\in R^{L\times L}$ act as gates, operating element‐wise on the projections. These projections are generated through the application of a dense linear layer followed by short convolutions on the input sequence. Upon obtaining the filter values and projecting the input signal, we utilize the fast fourier transformation (FFT) method, which operates at a computational complexity of $O(L\log_{2}\;L)$ in the frequency domain.

After the Hyena block, we incorporate a multilayer perceptron (MLP) decoding header. During the pre‐training phase, predicts the subsequent token for each input amino acid token via a softmax function. During the fine‐tuning phase, for token‐level tasks, each token is associated with a label output. Conversely, for sequence‐level tasks, the final embeddings of all tokens are averaged and processed through a softmax function to predict the label. This structured approach allows for precise predictions at both the granularity of individual tokens and the broader context of entire sequences, facilitating versatile applications in protein sequence analysis.

### Pre‐training ProtHyena

For pre‐training our ProtHyena, we adopt the base configuration as per the guidelines in [48]. Our setup starts with 2 Hyena layers with a recursion order of N=2. The model's embedding size is set to 256, and it contains 1024 feed‐forward hidden units. The batch sizes to 256, and during training, we maintain a maximum protein sequence length of 1024 for ProtHyena. We use an autoregressive training framework similar to GPT [49] for ProtHyena, which involves pretraining the model to maximize the probability of predicting the next amino acid based on the sequence of preceding amino acids. Given a sequence of protein consists of amino acids $\left(a_{1},a_{2},\ldots ,a_{N}\right)$, we try to maximize the following probability:

$$P(a_{1},a_{2},\ldots ,a_{N})=\prod_{i=1}^{N}P(a_{i}|a_{1},a_{2},\ldots ,a_{i-1})$$

where $P\left(a_{i}|a_{1},a_{2},\ldots ,a_{i-1}\right)$ represents the conditional probability of amino acid $a_{i}$ given all preceding amino acids in a specific protein sequence.

### Pre‐training ProtGPT‐tiny and ProtGPT‐base

ProtGPT models utilize a conventional transformer decoder architecture, with self‐attention serving as a crucial component. When given a sequence x of length L and dimension D, each head of the self‐attention mechanism transforms $x\in R^{L\times D}$into an output $y\in R^{L\times D}$ using the self‐attention operator A(x):

$$A(x)=\mathrm{softmax}(xW_{q}W_{k}^{T}x^{T}),y=A(x)xW_{v}$$

where $W_{q},W_{k}$ and $W_{v}\in R^{D\times D}$ are learnable linear projections for the query, key, and value, respectively. The softmax and scaling function, denoted as softmax, is also applied. This mechanism allows the model to capture pairwise relationships among all tokens in the sequence, thereby understanding the global context.

ProtGPT‐tiny is composed of 2 transformer decoder layers. It matches ProtHyena in terms of embedding size and feed‐forward hidden units, equating to an identical amount of trainable parameters. In contrast, ProtGPT‐base is larger, with 8 transformer decoder layers, an embedding size of 512, and 2048 feed‐forward hidden units. We set batch size to 256, and during training, we maintain a maximum protein sequence length of 1024 for ProtGPT‐tiny. Due to memory constraints, the maximum length for ProtGPT‐base is limited to 512. Training is performed utilizing the Adam optimizer [50], starting with an initial learning rate of 0.0006 and employing a cosine decay learning schedule. The total number of training steps is approximately 30k.

### Model metric of pre‐training ProHyena, ProGPT‐tiny and ProtGPT‐base

We use perplexity to measure the effectiveness of our pre‐training on ProHyena, ProtGPT‐tiny and ProtGPT‐base. Perplexity is a measurement used in the field of natural language processing and, by extension, in protein language modeling, to evaluate the ability of probability models to predict a sequence. A lower perplexity score indicates that the model predicts the sample more accurately, implying a better performance. It is mathematically defined as the exponentiated average negative log‐likelihood of a protein sequence. If we consider a protein sequence of length L, with A representing the sequence of tokens (amino acids in the case of proteins), the perplexity PPL is computed using the following formula:

$$\mathrm{PPL}(A)=P{\left(a_{1},a_{2},\ldots ,a_{L}\right)}^{-\frac{1}{L}}$$

where $P(a_{1},a_{2},\ldots ,a_{L})$ is the probability of the sequence according to the model. This can be further broken down into:

$$\mathrm{PPL}(A)=\sqrt[{L}]{\frac{1}{P(a_{1},a_{2},\ldots ,a_{L})}}$$

In our case, for numerical stability and to simplify the computation, perplexity is calculated as the exponentiation of the cross‐entropy loss:

$$\mathrm{PPL}(A)=e^{H(A)}$$

where H(A) is the cross‐entropy of the sequence A, defined as:

$$H(A)=-\frac{1}{N}\sum_{i=1}^{L}\log P(a_{i}|a_{1},\ldots ,a_{i-1})$$

A lower perplexity score indicates a protein language model's enhanced learning of protein sequences.

For detailed hyperparameter settings for pretraining ProtHyena, ProtGPT‐tiny, and Prot‐base, please refer to Table S2. The hyperparameters for fine‐tuning are summarized in Tables S3‐S5.

### Finetuning for downstream tasks

For ProtHyena, ProtGPT‐tiny, ProtGPT‐base, and other comparison models, we extract embeddings from the protein sequences for downstream tasks. These embeddings are passed through a new decoder (a linear layer) to generate predictions, which are then compared with the labels to calculate the loss.

For residue‐level tasks, such as protein disorder and secondary structure prediction, the embeddings are tensors with dimensions corresponding to batch size, sequence length, and hidden dimension. The model generates predictions for each position in the sequence (batch size, sequence length). For protein‐level tasks, such as neuropeptide cleavage, signal peptide prediction, solubility, fluorescence, stability, and viral protein function, we average the embeddings across the sequence length to produce a single prediction for the entire protein.

### Metrics for downstream tasks

For the regression tasks (Fluorescence and Stability prediction), we evaluate model performance using Spearman correlation coefficient, Mean Squared Error (MSE), Mean Absolute Error (MAE), and Student *t* loss. Spearman correlation measures the strength and direction of the association between two ranked variables, MSE quantifies the average squared difference between observed and predicted values, MAE measures the average magnitude of errors, and Student's *t*‐loss considers the distribution of errors. For binary classification problems (Cleavage, signal peptide, Solubility, Disorder prediction), we report performance metrics such as Accuracy, F1 Macro Score, Matthew's Correlation Coefficient (MCC), and Area Under the Receiver Operating Characteristic Curve (AUROC) for each task. Accuracy measures the proportion of correct predictions, F1 Macro Score evaluates the balance between precision and recall. MCC is a metric well‐suited for evaluating models on imbalanced datasets, and it works for both binary and multi‐class classification problems. It considers true positives (TP), true negatives (TN), false positives (FP), and false negatives (FN), allowing it to assess the model's performance across all classes.

The value of MCC ranges from ‐1 to 1:

MCC = 1: The model's predictions are perfect, with no mistakes.

MCC = 0: The model performs no better than random guessing.

MCC = ‐1: The model's predictions are entirely wrong, with all positive samples predicted as negative and vice versa.

The formula to calculate MCC is:

$$\text{MCC}\;=\frac{\mathrm{TP}\times \mathrm{TN}-\mathrm{FP}\times \mathrm{FN}}{\sqrt{\left(\mathrm{TP}+\mathrm{FP}\right)\left(\mathrm{TP}+\mathrm{FN}\right)\left(\mathrm{TN}+\mathrm{FP}\right)\left(\mathrm{TN}+\mathrm{FN}\right)}}$$

AUROC represents the ability of the model to distinguish between classes. For the multiclass classification task (viral protein function prediction), we report the AUROC and AUPRC for each class, where AUROC indicates the model's performance across different threshold settings, and AUPRC measures precision‐recall trade‐off.

### Comparison to other protein language models

To assess ProtHyena, we also fine‐tune our pre‐trained ProtGPT‐tiny, ProtGPT‐base and other pre‐trained protein language model including ESM‐1b [41], ESM‐1v [51], ESM‐2 [40], ProtBert‐U100 [6], ProtBert‐BFD [6] and ProtT5‐XL‐U50 [6] in eight protein related downstream tasks. ESM‐1b and ESM‐2 are both 650 M parameter models based on the transformer encoder architecture and trained using a masked language modeling objective. The primary differences between these models lie in their training data and positional encoding methods. ESM‐1b is trained on 27.1 million UniRef50 [52] representative sequences and utilizes learnable sinusoidal encoding in the position embedding layer. In contrast, ESM‐2 is trained on 60 million protein sequences and employs rotary position embedding, which allows the model to extrapolate beyond the context window it is trained on. Additionally, ESM‐1v, which shares the architecture of ESM‐1b, is a 650 M parameter transformer language model specifically designed for predicting variant effects. It is trained on a diverse data set of 98 million protein sequences across various evolutionary lineages. ProtBert‐U100 and ProtBert‐BFD are both 420 M parameter models based on the BERT architecture. ProtBert‐U100 is trained on UniRef100 [52], which contains 216 million protein sequences, while ProtBert‐BFD is trained on BFD [53], which includes 2,122 million protein sequences. Additionally, ProtT5‐XL‐U50 is a model based on the T5 [54] architecture with 3 billion parameters, trained on UniRef50. In addition, we also apply one non‐attention based deep learning model (CNN) in fluorescence landscape prediction task. For the CNN, we use 35 residual blocks, each containing two convolutional layers with 256 filters, a kernel size of 9, and a dilation rate of 2, with a total of 41 million trainable parameters.

### Task definition and biological importance

#### Task 1: Fluorescence landscape prediction

**Definition:** This task is a form of regression analysis where each input, represented by a protein sequence, is assigned a numeric label that falls within the real number range. This label denotes the log‐fluorescence intensity of the protein [21].

**Biological Importance:** Considering a protein with length L, the total number of possible sequences that differ by m mutations is on the order of $O\left(L^{m}\right)$, which quickly becomes an infeasibly vast space for thorough exploration through experimental methods, particularly for even modest values of m. Furthermore, the presence of epistasis—meaning interactions among mutations at different positions that affect the outcome in second‐order or higher dimensions—makes it unlikely that straightforward, incremental optimization techniques will be effective. Accurate computational predictions could significantly streamline the process of navigating this complex landscape, leading to the discovery of more optimal solutions. Machine learning approaches have already demonstrated promise in similar tasks within the realm of protein engineering.

#### Task 2: Stability landscape prediction

**Definition:** This task is set as a regression where each input, denoted by a protein x, is associated with a numeric label y∈R. This real number label quantifies the most challenging conditions under which the protein can retain its structure above a specific concentration level, serving as an indicator of its intrinsic stability [45].

**Biological Importance:** The creation of stable proteins is crucial, especially in contexts like drug delivery, where it is essential that the drugs remain intact until they reach their target. On a broader scale, being able to sift through extensive protein data to pinpoint and refine the best candidates can significantly enhance the efficiency and output of costly protein engineering experiments, optimizing resources and yielding better experimental outcomes.

#### Task 3: Neuropeptide cleavage prediction

**Definition:** The task involves predicting whether each amino acid residue within a given protein sequence is a cleavage site. This process is essential for understanding how proteins are processed and activated through posttranslational modifications, particularly through the cleavage of precursor proteins into their functional forms [26].

**Biological Importance:** These cleavage processes are crucial for activating or deactivating the proteins and peptides involved in various cellular functions, including communication within the endocrine and nervous systems. The ability to predict these sites accurately aids in the analysis of new proteomes at a genomic scale, offering insights into protein function and the discovery of new bioactive peptides.

#### Task 4: Disorder prediction

**Definition:** The task focuses on analyzing a given protein sequence to classify regions as either “ordered” or “disordered”. Ordered regions are structured parts of the protein, whereas disordered regions lack a fixed three‐dimensional structure under physiological conditions. This classification is crucial for understanding protein function and interaction capabilities [27].

**Biological Importance**: Identifying disordered regions in proteins is key to understanding their roles in biological processes. Disordered regions often participate in signaling and regulation mechanisms within the cell due to their flexibility and ability to bind multiple partners. Recognizing these regions helps in predicting protein functions, understanding disease mechanisms, and identifying potential drug targets.

#### Task 5: Signal peptide classification

**Definition:** Signal peptide classification is a crucial task where each protein sequence is analyzed to identify a short segment at the amino terminus, known as a signal peptide (SP). These SPs guide the protein to or across membranes within the cell. SignalP 5.0 introduces a deep neural network‐based approach that not only predicts the presence of SPs but also distinguishes among three types of prokaryotic SPs, enhancing prediction across all domains of life.

**Biological Importance:** The detection and classification of signal peptides are vital for our understanding of protein targeting and membrane translocation mechanisms in cells. SPs direct proteins to their correct cellular or extracellular locations, which is fundamental for protein function and cellular organization. Accurately identifying SPs is especially crucial in microbiology and medicine, for example, in tracking the emergence of antibiotic‐resistant genes or discovering new enzymes for genome editing technologies. The ability to differentiate between various SP types improves our understanding of protein pathways and functions, contributing significantly to advances in biomedical research and therapeutic development.

#### Task 6: Solubility prediction

**Definition:** This is a binary classification task involves analyzing a given protein sequence to predict whether the protein is soluble.

**Biological Importance:** This prediction is vital for applications in biochemistry and pharmacology, where solubility influences protein expression, purification, and therapeutic efficacy. The task requires accurately assessing the protein's sequence to foresee its solubility status, facilitating further studies on its functional and therapeutic potential.

#### Task 7: Gene function prediction

**Definition:** This task is set as a classification task where each input, denoted by a protein x, is associated with a label y∈ {transcription regulation; tail; other; moron, auxiliary metabolic gene and host takeover; lysis; integration and excision; head and packaging; connector; DNA, RNA and nucleotide metabolism}.

**Biological Importance:** The creation of stable proteins is crucial, especially in contexts like drug delivery, where it is essential that the drugs remain intact until they reach their target. On a broader scale, being able to sift through extensive protein data to pinpoint and refine the best candidates can significantly enhance the efficiency and output of costly protein engineering experiments, optimizing resources and yielding better experimental outcomes.

#### Task 8: Secondary structure (SS) prediction

**Definition:** Predicting the secondary structure of proteins is a task that matches each amino acid in a sequence to one of three possible categories: Helix, Strand, or Other.

**Biological Importance:** Understanding a protein's secondary structure is crucial for determining its function, particularly when the protein in question has no evolutionary similarity to any proteins whose structure is already known. Secondary structure prediction tools are widely utilized to generate detailed input features for more complex modeling tasks.

### Manual

To help researchers effectively use ProtHyena, we provide pre‐trained weights along with fine‐tuned weights for various tasks. We also offer detailed instructions for fine‐tuning the model on specific tasks and guidelines for organizing new downstream datasets to support further fine‐tuning. For convenience, we provide a Colab notebook that allows inference across different downstream tasks without the need to set up a local environment. The notebook loads fine‐tuned models directly from Hugging Face. Researchers only need to select the desired model, set the decoder type and number of classes (for regression tasks, the number of classes is 1), and input the protein sequence for analysis. Further details can be found in our GitHub repository: https://github.com/ZHymLumine/ProtHyena.

## AUTHOR CONTRIBUTIONS

Yiming Zhang did the experiments, analyzed data, wrote the code and the manuscript. Bian Bian contributed to shaping the research, assisted the analysis, and revised the manuscript. Manabu Okumura supervised this project. All authors have read the final manuscript and approved it for publication.

## CONFLICT OF INTEREST STATEMENT

The authors declare no conflicts of interest.

## ETHICS STATEMENT

No animals or humans were involved in this study.

## Supporting information

Figure S1. Comparison of parameters between ProtT5‐XL, ESM, ProtBert, ProtGPT‐base, ProtGPT‐tiny and ProtHyena.
Figure S2. Label distribution across different protein property tasks.
Figure S3. Evaluation on Protein Secondary Structure Prediction.

Table S1. Summary of training time for six downstream tasks.
Table S2. Hyperparameter settings for ProtGPT‐tiny, ProtGPT‐base and ProtHyena pretraining.
Table S3. Hyperparameter settings for ProtHyena on mulit‐class classification tasks.
Table S4. Hyperparameter settings for ProtHyena on binary classification tasks.
Table S5. Hyperparameter settings for ProtHyena on regression classification tasks.

## Data Availability

The data that support the findings of this study are openly available in ProtHyena at https://github.com/ZHymLumine/ProtHyena. The code required to reproduce the results in this study is available at https://github.com/ZHymLumine/ProtHyena. Supplementary materials (figures, tables, scripts, graphical abstract, slides, videos, Chinese translated version and update materials) may be found in the online DOI or iMeta Science http://www.imeta.science/imetaomics.
