# Supplementary material for: Hyena architecture enables fast and efficient protein language modeling
Source: IMetaOmics. 2024 Dec 7;2(1):e45. doi: 10.1002/imo2.45 (PMC12806224; doi:10.1002/imo2.45)
Supplement: Supplementary file 1 — Figure S1. Comparison of parameters between ProtT5‐XL, ESM, ProtBert, ProtGPT‐base, ProtGPT‐tiny and ProtHyena. Figure S2. Label distribution across different protein property tasks. Figure S3. Evaluation on Protein Secondary Structure Prediction. [file IMO2-2-e45-s001.docx]

**Supporting information to**

**Hyena Architecture Enables Fast and Efficient Protein Language Modeling**

## **Running Title**: ProtHyena: Hyena Architecture in Protein Language Modeling

Yiming Zhang^1^, Bian Bian^2,3,4^, Manabu Okumura^1*^

^1^Department of Information and Communications Engineering, School of Engineering, Institute of Science Tokyo, Yokohama, Kanagawa 226-8503, Japan

^2^Department of Computational Biology and Medical Sciences, Graduate School of Frontier Sciences, The University of Tokyo, Kashiwa, Chiba 277-8561, Japan

^3^Artificial Intelligence Research Center, National Institute of Advanced Industrial Science and Technology (AIST), Koto-ku, Tokyo 135-0064, Japan

^4^Department of Data Science, School of Frontier Engineering, Kitasato University, Sagamihara, Kanagawa 252-0373, Japan

*Correspondence: [oku@pi.titech.ac.jp](file:///Users/zym/Downloads/Overleaf/ProtHyena/oku@pi.titech.ac.jp) (Manabu Okumura)

### **Enhanced Efficiency and Performance of ProtHyena in Protein Language Modeling**

In Table S1, we present the training time for one epoch across six tasks to assess efficiency in downstream tasks. The detailed parameter comparison among the other different protein language models used in this study is presented in Figure S1.

**Fine-tuning downstream benchmarks**

To assess ProtHyena, we incorporated eight benchmark datasets from previous protein language modeling studies, following the same data splits for consistency, and performed comprehensive performance evaluations across a variety of downstream tasks, as elaborated in Table S2. These evaluations span tasks at different resolutions, including single amino acid (residue) level and protein sequence level, covering both classification and regression tasks. This thorough assessment provides a holistic view of ProtHyena's performance. In Figure S2, we presented the label distribution for the four binary classification tasks. Notably, only the solubility task has relatively balanced labels. In the Neuropeptide Cleavage Prediction task, the ratio of 0 to 1 is approximately 3:7. For Disorder Prediction, almost all labels are 1. In the Signal Peptide Prediction task, the ratio of 0 to 1 is about 2:8. To better evaluate model performance on these imbalanced datasets, we use metrics such as macro F1, Matthew’s Correlation Coefficient (MCC), and Area Under the Receiver Operating Characteristic (AUROC). In predicting protein secondary structures, ProtHyena exhibited suboptimal performance compared to ESM [1] series, ProtBert and ProtT5-XL [2] models (Figure S3). Protein secondary structure prediction, being a residue-level task, involves a vast potential space of possibilities $3^{L}$ for a protein of length $L$, considering three structural categories per residue. In contrast, protein disorder prediction, also a residue-level task but binary, involves a significantly smaller search space of $2^{L}$. The exponential complexity of secondary structure prediction underscores the necessity for extensive data-driven learning and suggests that comprehensive learning of each residue's role through self-attention mechanisms is crucial.

**Pre-training and fine-tuning settings**

For pre-training our protein models, we adopted the base configuration as per the guidelines in [3]. Our setup starts with 2 Hyena layers with a recursion order of $N=2$. The model's embedding size is set to 256, and it contains 1024 feed-forward hidden units. To effectively demonstrate the Hyena operator's capabilities, we also trained two decoder-only transformer models, named ProtGPT-tiny and ProtGPT-base, for comparison.

ProtGPT-tiny is composed of 2 transformer decoder layers. It matches ProtHyena in terms of embedding size and feed-forward hidden units, equating to an identical amount of trainable parameters. In contrast, ProtGPT-base is larger, with 8 transformer decoder layers, an embedding size of 512, and 2048 feed-forward hidden units. We managed our batch sizes to 256, and during training, we maintained a maximum protein sequence length of 1024 for ProtHyena and ProtGPT-tiny. Due to memory constraints, the maximum length for ProtGPT-base was limited to 512. Training was performed utilizing the Adam optimizer [4], starting with an initial learning rate of 0.0006 and employing a cosine decay learning schedule. The total number of training steps was approximately 30k. Throughout pre-training, we used perplexity as our primary performance metric. We also provide the hyperparameter settings for ProtGPT-tiny, ProtGPT-base, and ProtHyena during pretraining in Table S2, the hyperparameter settings for ProtHyena on multiclass classification tasks in Table S3, the hyperparameters for binary classification tasks in Table S4, and settings for regression tasks in Table S5.

**Summary of the species of protein analyzed in various downstream tasks**

**Fluorescence prediction**: *Aequorea victoria*

**Stability prediction**: Natural proteins from *Homo sapiens*, and de novo computational designed proteins through oligo library synthesis and expressed in yeast *and* *Escherichia coli*.

**Neuropeptide cleavage prediction**: The data are sourced from NeuroPep database, which includes a total of 493 organisms, comprising 48 phyla from Chordata, 21 from Arthropoda, 12 from Mollusca, 5 from Annelida, 3 from Nematoda, 2 from Platyhelminthes, and 2 from Cnidaria.

**Disorder prediction**: The coat protein was derived from bacteriophage AP205, the C-terminal domain of gasdermin-B was from *Homo sapiens*, the receptor-binding domain of the glycoprotein was from the whitewater arroyo viru*s*, the DNA-binding protein was from *Aedes aegypti*, the LH3 hexon-interlacing protein was from snake adenovirus-I, the ice-binding protein was from Antarctica, UDP-glucose glycoproteinglucosyltransferase was from *Chaetomium thermophilm*, and the scaffoldin protein was from *Ruminococcus flavefaciens.*

**Solubility prediction**: *Escherichia coli*.

**Signal peptide prediction**: Four types of proteins: Sec/SPI, Sec/SPII, Tat/SPI, and ‘Other’ (globular proteins without SP and transmembrane proteins with an experimentally verified TM segment within the first 70 amino acids) are derived from Eukarya, Archaea, Gram-positive bacteria, and Gram-negative bacteria.

**Viral gene function prediction: The** data are sourced from PHROG v3, which includes 868,340 proteins from complete genomes of viruses infecting bacteria or archaea (2,318 from RefSeq and 2,669 from GenBank, April 2018), and 12,498 curated prophages derived from cultivated microbial isolates. Website: <https://phrogs.lmge.uca.fr/>

**Protein secondary structure prediction**: 12,185 crystal structures data are from Protein Data Bank and selected by the PISCES server. The species information is not mentioned in the original paper. The data are from PMID: 30785653.

## **REFERENCES**

1. Rives, Alexander, Joshua Meier, Tom Sercu, Siddharth Goyal, Zeming Lin, Jason Liu, Demi Guo, et al. 2021. “Biological structure and function emerge from scaling unsupervised learning to 250 million protein sequences.” *Proceedings of the National Academy of Sciences* 118: e2016239118. <https://doi.org/10.1073/pnas.2016239118>

2. Elnaggar, Ahmed, Michael Heinzinger, Christian Dallago, Ghalia Rehawi, Yu Wang, Llion Jones, Tom Gibbs, et al. 2022. “ProtTrans: Toward Understanding the Language of Life Through Self-Supervised Learning.” *IEEE Transactions on Pattern Analysis and Machine Intelligence* 44: 7112-7127. <https://doi.org/10.1109/TPAMI.2021.3095381>

3. Nguyen, Eric, Michael Poli, Marjan Faizi, Armin Thomas, Callum Birch-Sykes, Michael Wornow, Aman Patel, et al. 2024. “Hyenadna: Long-range genomic sequence modeling at single nucleotide resolution.” *Proceedings of the 37th International Conference on Neural Information Processing Systems* 43177 - 43201. <https://doi.org/10.5555/3666122.3667994>

4. Loshchilov, Ilya, Frank Hutter. 2017. “Decoupled weight decay regularization.” *arXiv preprint arXiv:1711.05101* <https://doi.org/10.48550/arXiv.1711.05101>

**Figure legends**

**Figure S1. Comparison of Model Parameters Across Different Protein Language Models.** The figure shows the number of parameters for various protein language models: ProtT5-XL (3 billion), ESM series (ESM-1b, ESM-1v and ESM-2) (650 million), ProtBert series (ProtBert-U100 and ProtBert-BFD) (420 million), ProtGPT-base (25.2 million), and ProtGPT-tiny & ProtHynea (1.6 million). The size of each circle represents the relative scale of parameters, providing a visual comparison of model complexities across these architectures.

**Figure S2. Label distribution across different protein property tasks.** The pie charts display the label distributions for both training and testing sets across four protein property prediction tasks: (A) Neuropeptide cleavage, (B) Disorder, (C) Signal peptide, and (D) Solubility. Each chart shows the proportion of samples labeled as 0 or 1 in both datasets, providing insight into the class imbalance present in each task.

## ****

**Figure S3. Evaluation on Protein Secondary Structure Prediction.** Accuracy, F1 macro score, and Area Under the Receiver Operating Characteristic Curve (AUROC) were used for ProtHyena and other protein language models.
